# Enriched Differentiation of Human Otic Sensory Progenitor Cells Derived From Induced Pluripotent Stem Cells

**DOI:** 10.3389/fnmol.2018.00452

**Published:** 2018-12-20

**Authors:** Hanae Lahlou, Emmanuel Nivet, Alejandra Lopez-Juarez, Arnaud Fontbonne, Said Assou, Azel Zine

**Affiliations:** ^1^LNIA, CNRS UMR 7260, Aix-Marseille Université, Marseille, France; ^2^Aix-Marseille Université, CNRS, INP UMR 7051, Marseille, France; ^3^IRMB, Université Montpellier, INSERM U1183, Montpellier, France; ^4^Université Montpellier, UFR de Pharmacie, Montpellier, France

**Keywords:** human otic progenitor cells, human induced pluripotent cells, otic development, embryonic hair cells, *in vitro* differentiation, transcriptome (RNA-seq)

## Abstract

Age-related neurosensory deficit of the inner ear is mostly due to a loss of hair cells (HCs). Development of stem cell-based therapy requires a better understanding of factors and signals that drive stem cells into otic sensory progenitor cells (OSPCs) to replace lost HCs. Human induced pluripotent stem cells (hiPSCs) theoretically represent an unlimited supply for the generation of human OSPCs *in vitro*. In this study, we developed a monolayer-based differentiation system to generate an enriched population of OSPCs via a stepwise differentiation of hiPSCs. Gene and protein expression analyses revealed the efficient induction of a comprehensive panel of otic/placodal and late otic markers over the course of the differentiation. Furthermore, whole transcriptome analysis confirmed a developmental path of OSPC differentiation from hiPSCs. We found that modulation of WNT and transforming growth factor-β (TGF-β) signaling combined with fibroblast growth factor 3 (FGF3) and FGF10 treatment over a 6-day period drives the expression of early otic/placodal markers followed by late otic sensory markers within 13 days, indicative of a differentiation into embryonic-like HCs. In summary, we report a rapid and efficient strategy to generate an enriched population of OSPCs from hiPSCs, thereby establishing the value of this approach for disease modeling and cell-based therapies of the inner ear.

## Introduction

Almost all cell types of the inner ear, including neurosensory, non-sensory and secretory cells derive from the otic vesicle, an epithelial structure that emerged through invagination of the otic placode (OP) during early organogenesis. Among developmental lineages in vertebrate embryo, the otic sensory lineage has the unique capacity to give rise to auditory and vestibular hair cells (HCs), supporting cells and neurons involved in both hearing and balance functions. Several signaling pathways including fibroblast growth factor (FGF), WNT and NOTCH are involved in the specification of OP as well as in otic sensory lineage in the embryo (Ohyama et al., 2006; Jayasena et al., 2008; Hartman et al., 2010; Hammond and Whitfield, 2011; Vendrell et al., 2013). At birth, the human inner ear contains about 75,000 sensory HCs (Lim and Brichta, 2016). Environmental insults such as loud noises and ototoxic drugs, genetic predisposition or aging, can each cause loss of HCs leading to permanent hearing loss or dizziness. Two approaches have been subjected to restore HCs that do not regenerate, i.e., gene and stem cell-based cell therapies (Géléoc and Holt, 2014; Zine et al., 2014). The stem cell approach requires the robust production of otic sensory progenitor cells (OSPCs) *in vitro* to provide material for cell grafting investigations in animal models of inner ear neurosensory degeneration. Over the past two decades, pluripotent stem cells (PSCs), either from embryonic origin or obtained by cell reprogramming (Takahashi et al., 2007), have received considerable attention for potential use of their derivatives in cell-based therapeutic applications. In the inner ear, several studies with murine embryonic stem cells (ESCs) or induced PSCs (iPSCs) have reported on the generation of otic progenitors in different cell culture models (Oshima et al., 2010; Koehler et al., 2013; Costa et al., 2015; Liu et al., 2016; Abboud et al., 2017), providing solid bases for establishing an *in vitro* platform with similar approaches whilst using human PSCs. In addition, certain culture protocols have shown the possible differentiation of human ESCs/iPSCs along HC and/or neuronal lineages (Chen et al., 2012; Ronaghi et al., 2014; Ealy et al., 2016) under embryoid body (EB)-based guidance and manipulation of molecular pathways that are active during inner ear development. Recently, the concept of differentiation of hESC-derived HC-like cells was elegantly demonstrated by the ability of self-guided differentiation of these cells when cultured in hydrogels as extracellular matrix for three-dimensional (3D) culture system (Koehler et al., 2017). Although EB-aggregates and 3D-organoids guidance methods can generate HC-like cells from PSCs, they have variable efficiencies, are time consuming and the early differentiation of human OSPCs remains elusive. Furthermore, the culture methods applied (EB and 3D organoids) in these studies are not the most appropriate for assessing the grafting-integration capacity of dissociated otic progenitors as they were focused on the generation of mature HC-like cells *in vitro*.

To address these limitations, we investigated the capacity of human induced PSCs (hiPSCs) to differentiate into otic/placodal progenitors without intervening multi-lineage aggregate or organoid intermediary steps, with the ultimate goal to derive bona fide human OSPCs. In this study, we focused on FGF, transforming growth factor-β (TGF-β) and WNT pathways, known for their crucial roles during inner ear ontogenesis. These pathways are involved in determining the size of OP and in the development of inner ear neurosensory components (Head et al., 2013; Jacques et al., 2013). We have established *in vitro* conditions for the rapid and efficient generation of human OSPCs from hiPSCs via a stepwise manipulation of FGF, TGF-β and WNT pathways through the use of a monolayer-based differentiation system. In this study, we applied qPCR and immunocytochemistry analyses to characterize the differentiated cells and we showed that our newly established differentiation protocol allows for the generation of human OSPC-like cells. These results were confirmed and further validated by RNA-seq analysis that revealed OSPC-like signature in those differentiated cells, as identified by a gene expression profile that matches with known otic sensory markers and major signaling pathways involved in otic development.

## Materials and Methods

### Cell Lines and Culture Conditions

The hiPSC lines (ChiPSC-4 and ASE-9202) used in this study were provided, respectively, by Cellartis (Göteborg, Sweden) and by Applied Stem Cell (Milpitas, CA, USA). The ChiPSC-4 cell line was derived from fibroblasts of healthy human donors and reprogrammed by using polycistronic retrovirus, based on the transduction of the following transcription factors: Oct3/4, SOX2, KLF4 and c-Myc (Takahashi et al., 2007). The other hiPSC line used in this study, namely the ASE-9202 line, was generated using non-integrating episomal vectors. Both pluripotent stem cell lines were maintained using two distinct feeder-free systems. ChiPSC-4 were plated at a density of 40,000 cells/cm^2^ onto dishes coated with a DEF-CS™ COAT-1 matrix (Cellartis), diluted at 1:20 in D-PBS (+/+; Life Technologies, Carlsbad, CA, USA). Cells were expanded in DEF-CS™ 500 basal medium, daily supplemented with DEF-CSTM GF-1 (1:333), GF-2 (1:1,000) and GF-3 (1:1,000) additives (Cellartis). When the cells were close to become confluent at 80%–90% (about 5–7 days), they were passaged using TrypLE Select^®^ (Life Technologies, Carlsbad, CA, USA). Pluripotency of reprogrammed cells was confirmed by qPCR and immunocytochemistry against pluripotent markers.

For the ASE-9202 hiPSC line, cells were cultured in chemically-defined StemMACS iPS-Brew medium (Miltenyi Biotec) on growth factor reduced Matrigel (Corning) coated plates. Briefly, 70%–80% confluent hiPSCs were treated with an enzyme-free solution (0.5 mM EDTA (Invitrogen, 15575-020), D-PBS (Invitrogen 14190-144) and 1.8 mg/mL NaCl (Sigma)) for 2 min at 37°C and the colonies were dispersed to small clusters and lifted carefully using a 5 mL glass pipette at a ratio of 1:4. Both cell lines were maintained in an incubator (37°C, 5% CO_2_) with medium changes every day.

### Otic Placode Induction and Otic Sensory Progenitor Differentiation

hiPSCs were dissociated to single cells using TrypLE Select^®^ (Life Technologies, Carlsbad, CA, USA) and plated on laminin-coated wells (1.5 μg/cm^2^; R&D Systems) at a density of 30,000 cells/cm^2^. For the first day of seeding (D0), cells were cultured in DFNB basal medium (DMEM/F12 with N2/B27) supplemented with fibroblast growth factors, FGF3 (50 ng/mL), FGF10 (50 ng/mL; R&D Systems) and 10 μM of ROCK inhibitor Y-27632 (StemCell Technologies) as established in our previous study (Lahlou et al., 2018). From day 1 of differentiation, the medium was replaced with induction medium: DFNB medium supplemented with 50 ng/mL FGF3, 50 ng/mL FGF10, 10 μM SB431542 (TGFβ inhibitor, Stemgent) and 100 ng/mL of recombinant human Dickkopf-related protein 1 (rhDKK1; WNT inhibitor, R&D Systems). Medium was replaced every other day until day 6. Then, the medium was supplemented with 50 ng/mL recombinant human WNT3A (WNT agonist, R&D Systems) instead of SB431542 and rhDKK1. Medium was replaced every other day until day 13.

### RNA Processing and qRT-PCR

Total RNA was extracted from hiPSCs and differentiated cells using the PureLink^®^ RNA Mini Kit (Life Technologies, Carlsbad, CA, USA) according to manufacturer’s instructions. cDNA was synthesized from 1 μg of RNA per sample, using High-Capacity RNA-to-cDNA™ Kit (Life Technologies, Carlsbad, CA, USA). cDNA was diluted 10-fold in DNA suspension buffer (Teknova) and used for the Fluidigm pre-amplification step for Dynamic Array gene expression analysis: cDNA was pre-amplified for 14 cycles with 500 nM DELTAgene pooled primer mix using 2x Taqman PreAmp Master Mix (Invitrogen), followed by Exo1 treatment (NEB). Five-fold diluted Exo1 treated pre-amplified cDNA was used for loading the 96.96 Dynamic Array chip on the Fluidigm Biomark HD. Primer pairs used for gene expression analysis are listed in Supplementary Table S1. The data were analyzed with Real-Time PCR Analysis Software in the BioMark instrument. Ct values were processed by automatic threshold for all assays, with derivative baseline correction using BioMark Real-Time PCR analysis Software 4.1.3 (Fluidigm). Statistical significance for relative fold change values was determined using Student’s *t*-test.

### Immunocytochemistry and Imaging Analysis

Differentiated cells were fixed with 4% paraformaldehyde in phosphate-buffered saline (PBS) for 20 min at RT. Unspecific binding was blocked in 0.3% Triton X-100 and 3% bovine serum albumin in PBS for 30 min at RT. The fixed cells were incubated overnight at 4°C with diluted specific primary antibodies listed in Supplementary Table S2. Then, the cells were washed, and incubated with the corresponding AlexaFluor secondary antibodies listed in Supplementary Table S2 and nuclei counterstained with Hoechst (1:1,000, Sigma-Aldrich). Control experiments including negative controls without primary antibodies were processed in parallel. Samples were mounted using Prolong Gold antifading (Life Technologies, Carlsbad, CA, USA) on glass slides. The images were acquired with Zeiss Axioimager/LSM 5 Exciter fluorescence or LSM 710 NLO Zeiss confocal microscope and the Zen software (Zeiss, Germany).

### Whole Transcriptome Analysis by RNA-Seq

Total RNA was extracted from cells using the PureLink^®^ RNA Mini Kit (Life Technologies, Carlsbad, CA, USA) according to manufacturer’s instructions. RNA samples were obtained from undifferentiated hiPSCs, differentiated cells at day 6 and day 13. Briefly, the RNAseq libraries were constructed according to the TruSeq Stranded (Illumina) protocol. We first purified and fragmented 500 ng of total RNA. We then synthesized cDNA, adenylated the 3′ ends and added adapters. We amplified the cDNA by PCR. The obtained libraries were controlled with LabChip GX Touch microfluidics (Perkin Elmer). All libraries were validated, then normalized and pooled. Finally, sequencing and base calling were performed using the Illumina MiSeq platform (approved ISO9001:2008), using the Paired-end reads 2 × 150 bp method, and sequences were obtained after purity filtering. Hierarchical clustering was produced using the Cluster and Treeview softwares (PMID: 9843981). To uncover functional biological networks, we imported gene expression signatures into the Ingenuity Pathways Analysis (IPA) Software^1^.

Gene expression data from this study has also been deposited at ArrayExpress database under accession number E-MTAB-6679.

### Cell Counting and Statistical Analysis

The cells were counted manually using graphic tools of the Zeiss computer software (Zen 2012). The fraction of immuno+ cells among the total number of cells identified by Hoechst staining was determined in ten fields per coverslip in each *in vitro* condition and for each replicate experiment. Each field to analyze was randomly chosen to avoid any experimental bias. Three independent experiments were conducted for each determination and data were expressed as mean ± SEM (standard error of the mean). Throughout the study, the “*N*” represents the number of experiments performed independently, at different time, using different cell passaging with the same protocol (technical replicates). In addition, and for each experiment, every condition was run in triplicate for Fluidigm (biological replicates with two independent lines). Graph Pad Prism software was used for statistical analysis. Data were analyzed using Student’s *t*-test or one-way ANOVA. Differences between means were tested by Mann-Whitney test. Statistical difference was reported for *p*-values below 0.05, an asterisk indicates significant differences between means (*p < 0.05; **p < 0.01; ***p < 0.001).

## Results

### Differentiation of hiPSC-Derived Early Otic/Placodal Progenitors

During otic development, many markers including non-neural ectoderm (NNE), preplacodal ectoderm (PPE) and otic lineage genes are expressed in a fluctuating manner. Some early otic genes are transiently down-regulated and later re-expressed, whereas, other genes are only transiently expressed or are continuously up-regulated during embryonic development.

Despite the unique nature of the otic sensory lineage, there are no known markers that unambiguously discriminate this lineage with a clear chronological identification of cell subpopulations during otic development in an *in vitro* environment.

Hereafter, we therefore used the term “otic/placodal progenitors” to refer to early otic/placodal cells expressing NNE/PPE and OP markers, whereas cells expressing markers of later development (i.e., *ATOH1*, *POU4F3, ‥)* known to have an embryonic expression during *native HC early differentiation* referred to them as initial HCs.

**Figure 1:**
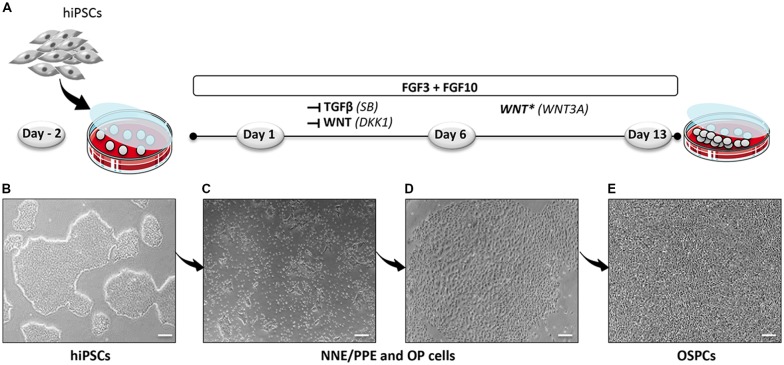
Optimized protocol for the differentiation of hiPSCs into hOSPCs. **(A)** Schematic illustration outlining the timeline and conditions of the newly established protocol for the generation of hiPSCs-derived hOSPC-like cells. **(B)** Representative bright field photograph of hiPSCs 2 days before differentiation (day-2) and grown as adherent colonies. **(C,D)** Representative bright field photographs, at day 6 of differentiation, that display differentiated colonies containing NNE/PPE and OP like-cells upon exposition to FGF3/10 along with a dual inhibition of TGF-β/WNT by applying both SB and DKK1 treatments. **(E)** A bright-field photograph of differentiated hOSPC-like cells at day 13 of differentiation according to the protocol described in **(A)**. Abbreviations: hiPSCs, human induced pluripotent stem cells; NNE, non-neural ectoderm; PPE, preplacodal ectoderm; OP, otic placode; hOSPCs, human otic sensory progenitor cells; FGF, fibroblast growth factor; TGF-β, transforming growth factor-β; SB-431542, TGF-β pathway inhibitor; DKK1, Dickkopf-related protein-1, WNT pathway inhibitor. Scale bars = 200 μm **(B–E)**.

**Figure 2:**
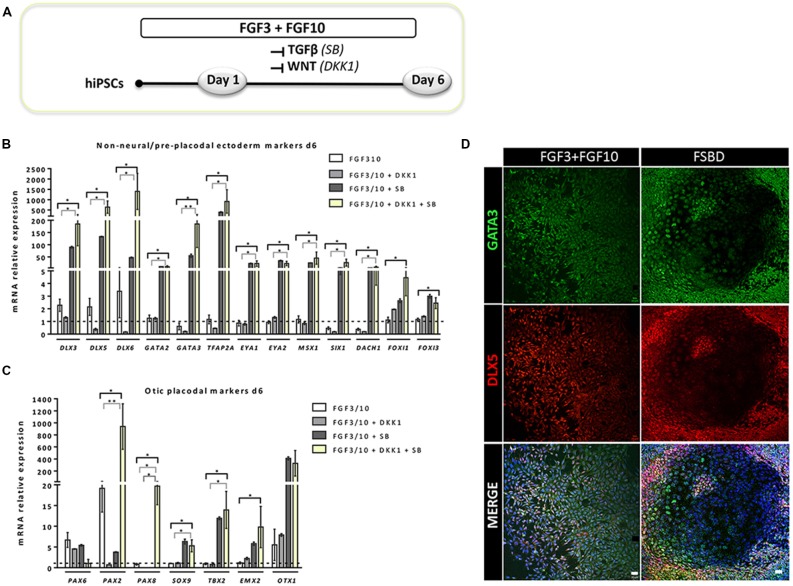
Rapid and efficient induction of early otic lineage markers in hiPSCs ongoing differentiation under TGF-β/WNT inhibition along with FGF treatment. **(A)** Scheme illustrating the first step of the established protocol (namely FSBD condition), which corresponds to a 6-day induction period as described. **(B,C)** Bar charts showing the relative gene expression levels obtained by qPCR analyses for two distinct panels of genes featuring the NNE/PPE **(B)** and OP **(C)** otic lineages, respectively. Cells were collected at day 6 of differentiation for the optimized FSBD protocol **(A)** and all control culture conditions (FGF3/10; FGF3/10+SB; FGF3/10+DKK1) and analyzed to assess the impact of the dual TGF-β/WNT inhibition on the otic differentiation. Noticeably, results demonstrate a significant upregulation of NNE/PPE and OP markers in the FSBD condition, which is the condition that yielded optimal otic induction from hiPSCs. Statistically significant differences between groups are indicated by *p < 0.05 and **p < 0.01 (*n* = 4). **(D)** Representative images showing DLX5 (red) and GATA3 (green) immuno+ cells at day 6 of differentiation in different condition with (FSBD, right) or without TGF-β/WNT inhibition (FGF3/10, left). Cell nuclei were counterstained with Hoechst (blue). Scale bars = 200 μm **(D)**.

To establish a protocol that enables the generation of human OSPCs by stepwise differentiation of hiPSCs, we devised a strategy that starts from adherent monolayer hiPSC cultures (Figure 1). According to previous studies, FGF3/10 treatment was necessary to initiate early otic differentiation from hiPSCs (Chen et al., 2012; Lahlou et al., 2018). Therefore, starting with FGF3/10-supplemented differentiation medium, we decided to test the impact of modulating pathways involved during otic development that may lead to a state in which hiPSCs would progressively differentiate and acquire an otic-like cells signature through a stepwise manner i.e., first to generate otic/placodal cells prior to generating hOSPCs. Following such a strategy and considering TGF-β and WNT pathways inhibition as two major events during early otic development, undifferentiated hiPSCs were first treated with SB431542 (i.e., a TGFβ inhibitor) and DKK1 (i.e., a WNT inhibitor) until day 6 of differentiation. To mimic WNT reactivation during the course of otic development, cultures were then challenged with WNT3A for an additional period of 7 days, until day 13. During the course of this 13-day differentiation protocol, the culture medium was continuously supplemented with FGF3/10 growth factors. As aforementioned, we designed experimental procedures to test and evaluate the impact of modulating TGF-β and WNT pathways in a sequential order as well as to evaluate a possible synergistic action. In order to identify the differentiation procedure that leads to the highest yields of OSPCs-like cells, we systematically tested four *in vitro* conditions as follows: FGF3/10+DKK1; FGF3/10+SB and FGF3/10+SB+DKK1 (hereafter referred as FSBD, Figure 1A). Daily observations of cells undergoing differentiation upon FSBD treatment revealed that hiPSCs underwent rapid and profound morphological changes, suggesting their progressive differentiation during the time course *in vitro* (Figures 1B–E). Noticeably, morphological changes were also observed with the other three conditions (not shown). To specifically assess the fate of differentiated cells during the early phase of differentiation, cells were either collected or fixed at day 6 and analyzed by qPCR and immunocytochemistry, respectively, for specific cell type markers of otic lineage specification i.e., NNE, preplacodal ectoderm (PPE) and OP cells (Figure 2). For all our culture conditions we explored the dynamic expression of a comprehensive panel of preselected NNE/PPE and OP known markers. Noteworthy, qPCR analyses showed that both FGF3/10+SB and FSBD differentiating conditions led to the robust expression of NNE/PPE markers, such as *DLX3/5/6*, *GATA2/3*, *EYA1/SIX1* (Figure 2B). Notably, in the FSBD condition the levels of expression of NNE/PPE associated genes were overall significantly higher when compared to other conditions. During inner ear development, NNE/PPE is swiftly followed by OP stage. Interestingly, OP marker expression analysis as early as 6 days of differentiation demonstrated the significant upregulation of a subset of genes such as, *PAX2/8*, *EMX2* and *SOX9* which are among key otic/placodal markers. Importantly, this significant upregulation of the OP-associated genes (i.e., *PAX2*, *PAX8*) was specific to the FSBD differentiating condition (Figure 2C). Strikingly, *PAX8* expression, which plays a critical role during OP induction together with *PAX2*, was only observed in cultures that were treated by FSBD. The differentiation seemed to be well driven upon FSBD treatment as other non-otic related genes such as *PAX6* were not overexpressed, which was not the case for other conditions, then suggesting the possible occurrence of other cell lineage contaminants in other conditions. Altogether these data indicate that, 6 days after differentiation in FSBD, hiPSCs underwent a rapid differentiation that led to the generation of a mixed population of cells acquiring gene expression profiles representative of cells at different stages of otic development i.e., NNE/PPE- and OP-like cells. Moreover, these results revealed that TGF-β inhibition along with FGF3/10 treatment contribute to the acquisition of cells expressing NNE/PPE markers whereas WNT inhibition seems to potentiate this differentiation and, of utmost importance, drives the differentiation of a fraction of cells toward the OP lineage within a 6-day period. To further validate our observations, we examined the expression of NNE/PPE markers by immunocytochemistry in FSBD treated cells at 6 days of differentiation. To that end, we selected a set of transcription factors belonging to DLX and GATA families, reported as important in defining early otic/placodal identity (Leung et al., 2013; Ealy et al., 2016). Noticeably, we observed a large subset of cells expressing GATA3 and DLX5 in distinctive and overlapping patterns (Figure 2D).

Our cultures harbored also immuno-positive cells expressing the SOX2, a transcription factor important for self-renewal and pluripotency of ESCs/iPSCs as well as a marker for otic lineage cells that adopt prosensory identity (Kiernan et al., 2005b; Dabdoub et al., 2008). We used immunostaining to examine the co-expression of SOX2 and PAX2, to define the otic progenitors at a cellular level after the inductive phase at day 6 of differentiation in FGF3/10 and FSDB culture conditions. We observed a qualitative relative difference in the apparent immunostaining intensity where strongly expressing SOX2-positive cells were observed in FSDB cultures when compared to a moderate-to-low levels expressed in FGF3/10 cultures (Supplementary Figure S1). We found a subset of SOX2-expressing cells, in turn, expressed PAX2 indicating that it is likely that this subset of PAX2/SOX2 immuno+ cells represents the otic/placodal progenitor cells.

Altogether, these data from qPCR and immunohistochemistry analyses demonstrate that the differentiated cells from hiPSCs have been rapidly engaged toward otic/placodal cell fate by day 6 upon FSBD treatment.

### Enrichment of hiPSCs-Derived Cells Expressing OSPC and Embryonic HC Markers

**Figure 3:**
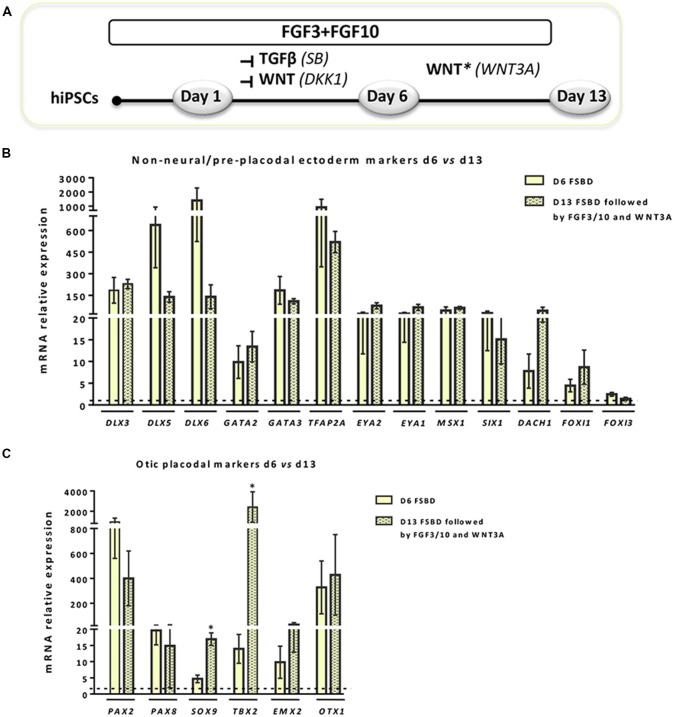
WNT activation impacts the expression of NNE/PPE and OP lineage markers in early-induced otic-like cells. **(A)** Scheme illustrating the second step of the newly established protocol, which corresponds to a 6-day induction period (FSBD) followed by the removal of SB and DKK1 compounds and replaced by WNT activation upon exposure to WNT3A with FGF3/10 for seven additional days. **(B,C)** Bar charts showing the relative gene expression levels obtained by qPCR analyses for two distinct panels of genes featuring NNE/PPE **(B)** and OP **(C)** otic lineages, respectively. According to the procedure described in **(A)**, cells were collected at day 6 and day 13 of differentiation in order to assess the impact of WNT activation on the further differentiation of early FSBD-induced cells (day 6). Of note, and even though differentiated cells maintained high gene expression levels for most OP markers, WNT3A treatment induced significant increase of some otic markers (i.e., *SOX9*, *p* = 0.028; *TBX2*, *p* = 0.035) when compared to their counterparts at day 6 of differentiation. Statistically significant differences between conditions are indicated by *p < 0.05 (*n* = 4).

**Figure 4:**
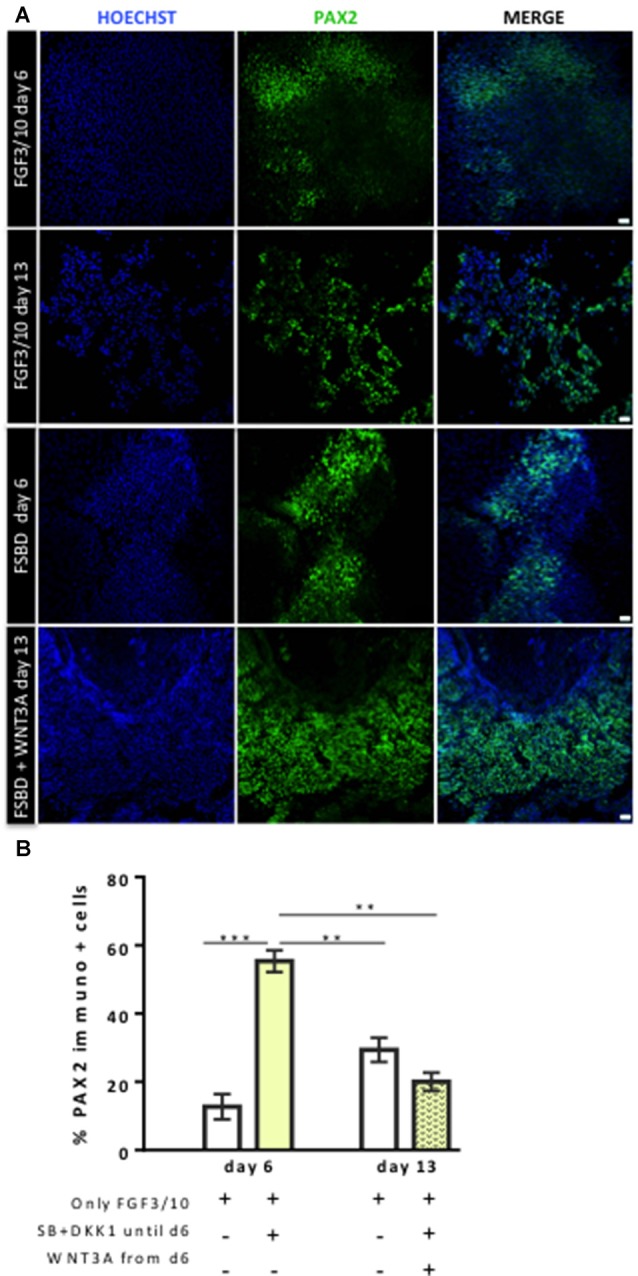
Dynamic expression of PAX2-expressing otic/placodal during the course of differentiation.** (A)** Representative images showing PAX2 (green) immuo+ cells in differentiated cultures obtained with two different conditions: FGF3/10 exposure for 13 days; or FSBD (6 days) followed by SB/DKK1 removal +WNT3A (7 days). Cell nuclei were counterstained with Hoechst (blue). **(B)** Quantitative analysis revealed a greater number of PAX2+ cells, at day 6 of differentiation, in differentiated cells obtained with the FSBD protocol. Of note, the number of PAX2+ cells obtained upon FSBD induction was significantly decreased at day 13, after WNT activation. Statistically significant differences between conditions are indicated by **p < 0.01 and ***p < 0.001 (*n* = 3). Scale bars = 200 μm.

**Figure 5:**
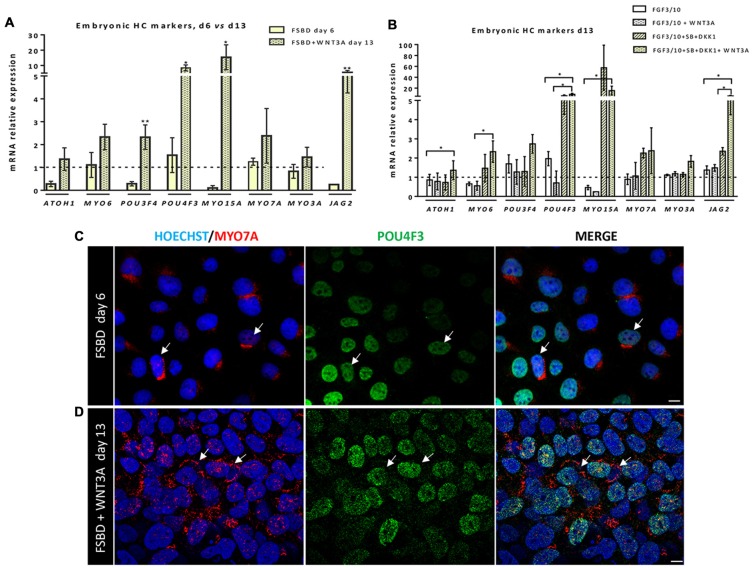
Early otic progenitors differentiate into cells expressing embryonic hair cell (HC) markers. **(A,B)** Bar charts showing the relative gene expression levels obtained by qPCR analyses for a panel of embryonic HC gene markers. In **(A)**, results indicate a significant increased expression of a subset of embryonic HC genes (e.g., *POU4F3, MYO15A, JAG2*) in cells at day 13 of differentiation, after WNT3A exposure, when compared to day 6 FSBD cultures. In **(B)** qPCR analyses at day 13 of differentiation show a significant increase in the expression of some embryonic HC (*ATOH1, MYO6, JAG2*) markers in FSBD followed by WNT3A-treated cells when compared to other culture conditions as indicated. Statistically significant differences between groups are indicated by *p < 0.05 and **p < 0.01 (*n* = 4). **(C)** Representative images showing POU4F3 (green) and MYO7A (red) immunostaining on cells that were fixed at day 6 in FSBD and at day 13 in FSBD followed by WNT3A **(D)** *in vitro* differentiating conditions. Interestingly, a subset of differentiated cells displayed co-expression of MYO7A mainly at a perinuclear cap-like structure (shown in red) and POU4F3 in the nucleus (shown in green). The arrows show POU4F3/MYO7A immuno+ cells. The cell nuclei were counterstained with Hoechst (blue). Scale bars = 100 μm.

Previous studies have demonstrated a role of WNT signaling pathway in otic development *in vivo* (Ohyama et al., 2006; Chai et al., 2012). It can also enhance the production of mammalian inner ear organoids (DeJonge et al., 2016; Koehler et al., 2017). Thus, we tested whether prolonging the culture period under WNT activation could promote the cells into more differentiated otic lineage cells. For this purpose, otic/placodal progenitors were treated with either FGF3/10 alone or in combination with WNT3A (as a recombinant protein) during the second step of *in vitro* differentiation (from day 6 to day 13; Figure 3A). Differentiated cells were harvested from these two culture conditions to assess their phenotypic profiles by qPCR and immunocytochemistry analyses. At day 13, cells that were challenged with FSBD+WNT3A showed a slight decrease in the relative expression levels of a subset of NNE/PPE-associated genes in differentiated cultures between day 6 and day 13 *in vitro*. Nevertheless, we found statistically significant variations in the relative expression levels of some OP-associated genes (e.g., *SOX9 p* = 0.028; *TBX2 p* = 0.035) indicative of a possible phenotypic change (Figures 3B,C). Noticeably, a trend of *PAX2* decrease was also observed, though not statistically significant. To gain further insights into the phenotypic status of differentiated cells at day 6 and day 13, we quantified the number of PAX2 immuno+ cells in the different culture conditions (Figures 4A,B), with PAX2 being considered among one of the most robust otic markers in mouse inner ear development (Burton et al., 2004; Christophorou et al., 2010). At day 6, we found that 55% (±3%, ***p = 0.008) of cells differentiated in FSBD were PAX2 immuno+ whereas only 13% (±2%) PAX2+ cells were found in the FGF3/10 culture condition. Remarkably, the number of PAX2+ cells in the FSBD treated cells was significantly decreased following WNT3A treatment (Figure 4B). These PAX2 immuno+ cell quantifications suggest that a subset of differentiating cells was rapidly engaged toward otic lineage as early as day 6 (i.e., FSBD condition) and potentially able to initiate expression of late OSPC markers (i.e., embryonic HCs). However, we cannot here rule out the possibility that this decreased could have also happened without WNT3A treatment. Nevertheless, these data provide important information regarding the distinct dynamic expression of PAX2+ cells in our newly defined differentiating conditions when compared to the FGF3/10 differentiating conditions. To further determine whether WNT activation of FSBD-induced cells was able to enhance a late OSPC phenotype, we determined the expression of a panel of known embryonic HC markers. Overall, qPCR results demonstrated an upregulation in the relative expression levels of embryonic HC gene markers in differentiated cultures at day 13 when compared to their expression levels at day 6 *in vitro* (Figure 5A). Thus, we noticed a significant increase in the relative gene expression levels of a subset of embryonic HC markers (i.e., *MYO15A*, *POU4F3*, *JAG2*) in FSDB/WNT3A cultures at day 13 (Figure 5A). In addition, comparative analyses of differentiated cells at day 13 revealed a significant increase in the expression levels of a subset of embryonic HC-associated genes (*ATOH1*, *POU4F3*, *MYO6*, *MYO15A* and *JAG2*) only in those cultures that were initially induced in FSBD condition during the first 6 days. Indeed, we showed that FGF3/10 treatment alone, with or without WNT3A induction starting from day 7, was not able to induce an embryonic HC-gene signature within 13 days of *in vitro* differentiation. Of importance, however, we found that this increase of embryonic HC-associated genes in FSBD-treated cells was independent of the absence or presence of WNT3A in the culture medium from day 7 (Figure 5B). Nevertheless, most of these early embryonic HC markers were found expressed at higher levels in the FSBD/WNT3A differentiating condition, suggesting that WNT3A treatment of FSBD-treated cells can facilitate the transition into an embryonic HC-like phenotype. Since POU4F3 and MYO7A are considered among key embryonic HC markers (Sahly et al., 1997; Hertzano et al., 2004), we next examined their presence by immunocytochemistry in differentiated cells at day 6 (FSBD) and day 13 (FSBD+WNT3A) culture conditions. Interestingly, POU4F3 and MYO7A immuno+ cells were found at day 6 (Figure 5C) and day 13 cultures (Figure 5D), and a subset of cells were found to co-express these embryonic HC markers.

**Figure 6:**
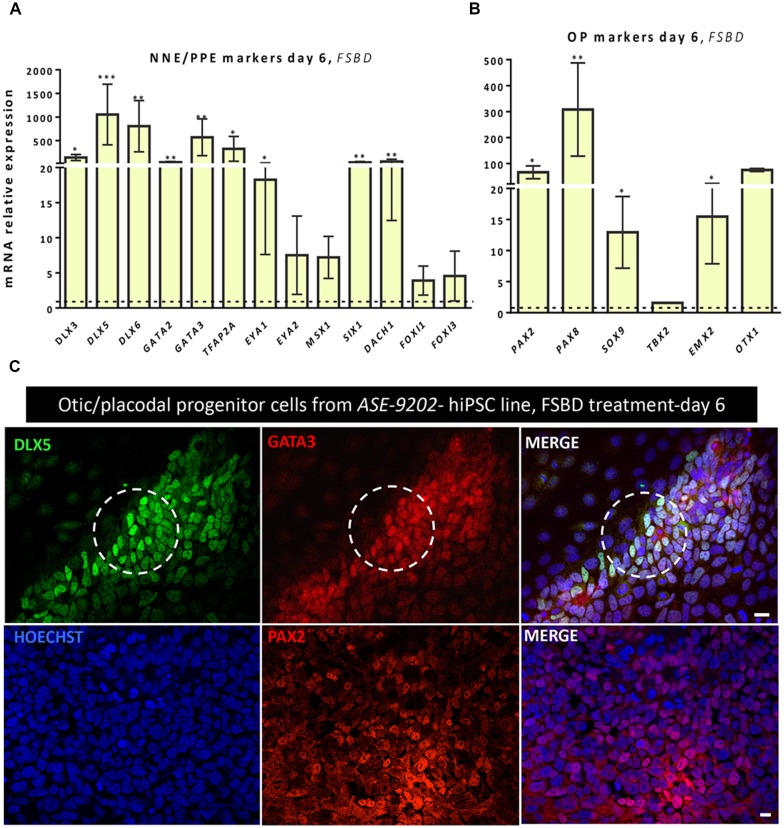
Analyses of NNE/PPE and OP markers differentiated cells from another hiPSC line reveal the robustness and reproducibility of the newly established protocol. An additional hiPSC line (ASE-9202) was subjected to the protocol we optimized on the ChiPSC-4 hiPSC line (see Figure 1A). Similarly to what we observed with the ChiPSC-4 hiPSC line, we observed a significant increase in the expression of a panel of genes representative of the NNE/PPE **(A)** and OP **(B)** lineages at day 6 of differentiation (FSBD culture condition). Relative gene expression levels were compared to the levels found in their undifferentiated counterparts. Statistically significant differences between groups are indicated by *p < 0.05, **p < 0.01 and ***p < 0.001 (*n* = 5).** (C)** Representative images showing the presence of numerous DLX5 (green, upper row) and GATA3 immuno+ cells (red, upper row) after 6 days of FSBD treatment as well as the presence of PAX2 (red, lower row) immuno+ cells. The circles indicate areas with a subset of DLX5/GATA3 immuno+ cells. Cell nuclei were counterstained with Hoechst (blue). Scale bars = 100 μm **(C)**.

In order to check the reproducibility and the robustness of our *in vitro* otic differentiation model, we tested the same *in vitro* differentiation paradigm using another hiPSC line. Quantitative PCR analysis showed similarities in the results from the two hiPSC lines used in this study. Thus, we found a significant upregulation in the gene expression levels of a subset of known NNE/PPE (i.e., *DLX5/6*, *GATA3*) and OP (i.e., *PAX2/8*, *SOX9*) markers at day 6 in FSBD cultures (Figures 6A,B) as what we observed with the initially tested hiPSC line (i.e., CHIPC-4) under similar culture conditions. In addition, immunocytochemistry analyses for some representative known lineage markers confirmed the robust expression of NNE/PPE (GATA3, DLX5) and OP (PAX2) in differentiated cells at day 6 in FSBD-treated cultures (Figure 6C).

### Transcriptome Analysis Delineates a Trajectory Toward hOSPC Lineage

**Figure 7:**
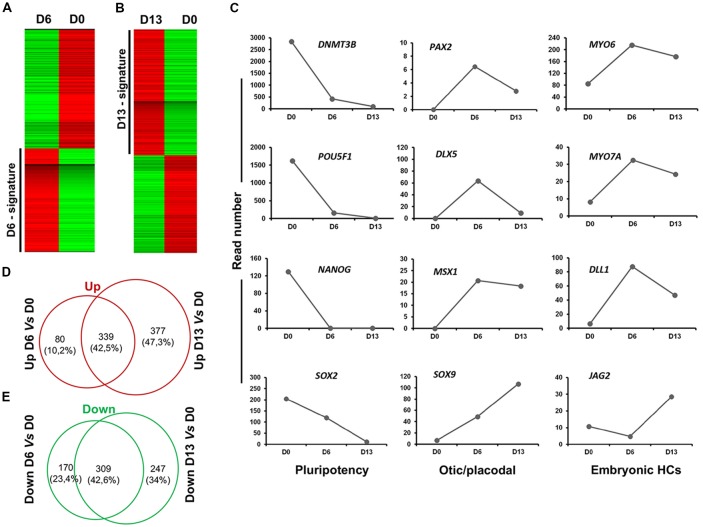
Analysis of cell lineage trajectories by RNA-seq profiling of hiPSCs derived OSPCs. **(A,B)** Heat maps. The molecular signatures of differentiated cells at D6 vs. hiPSCs at day of seeding (D0) and differentiated cells at D13 vs. hiPSCs at D0 were visualized by hierarchical clustering based on the differential expression transcripts. Transcripts are arranged in rows and the cell samples are arranged in columns. Red and green indicate expression above and below the mean, respectively, and white represents the mean expression. **(C)** The figure shows read numbers of 12 selected lineage-specific gene markers that are gradually modulated during the time course of otic induction guidance protocol. RNA-Seq-based transcriptomic revealed: (i) a drastic downregulation of the hiPSCs pluripotency genes during successive differentiation processes; and (ii) an upregulation of a subset of known otic/placodal and embryonic HC stage gene markers at three different time points of differentiation. **(D,E)** Venn diagram detailing shared and distinct up and down regulated genes among D13 and D6 in comparison to undifferentiated hiPSCs. D0, day 0; D6, day 6; D13, day 13 *in vitro*.

To further characterize and confirm the identity of enriched genes in early (day 6) and late (day 13) otic lineage cell populations, we decided to perform RNA-seq analysis on differentiated cells generated with our newly established *in vitro* protocol. Supervised hierarchical clustering of the transcriptomic data of hiPSCs and differentiated cells showed that undifferentiated hiPSCs at day 0 were clearly distinct from the differentiated otic/placodal progenitors in FSBD condition samples harvested at day 6 (Figure 7A) and in FSBD/WNT3A condition samples at day 13 (Figure 7B), respectively. The hiPSC signature included known pluripotency genes, such as *POU5F1*, *NANOG*, *SOX2* and *DNMT3B*. Conversely, the differentiated day 6 and day 13 signatures were characterized by the expression of genes, such as *PAX2*, *DLX5, MSX1* and *SOX9* that are involved in otic/placodal determination as well as of genes that are important for embryonic HC initial differentiation, such as *MYO6*, *MYO7A*, *DLL1* and *JAG2*. The expression kinetics of these genes in different samples indicated that pluripotency marker expression decreased during a progression to the early otic/placodal lineage while, a panel of OSPC and embryonic HC gene markers were enriched in differentiated cells at day 13 differentiated cultures (Figure 7C). For example, *DLX5*, *MSX1* and *PAX2* exhibited higher levels at day 6 and then decreased at day 13 while *SOX9* exhibited a progressive increase from day 0 to day 13. Interestingly, embryonic HC gene markers, (i.e., *JAG2*) is markedly enriched in differentiated cells at day 13 cultures. The gene overlap in day 6 and day 13 differentiated cells as compared to undifferentiated hiPSCs at day 0 was identified using a Venn diagram. Pairwise analyses indicated that of all differentially regulated genes about 42% of up and down-regulated genes are shared between day 6 and day 13 and that day 13 cell population contains more specific genes (Figures 7D,E).

**Figure 8:**
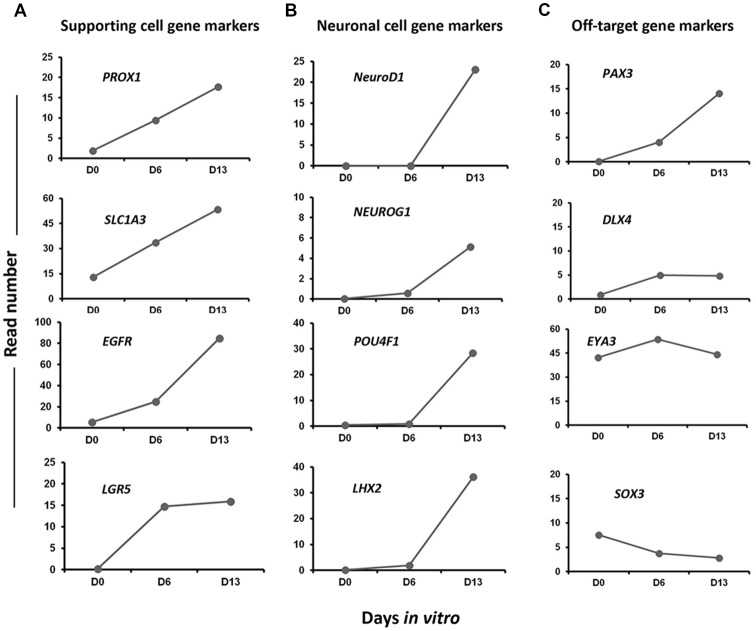
Expression profiles of selected gene markers in Day 0, Day 6 and Day 13. The figure shows read numbers of **(A)** supporting cell gene markers (*PROX1*, *SLC1A*3, *EGFR*, *LGR5*), **(B)** neuronal cell gene markers (*NEUROD1*, *NEUROG1*, *POU4F1*, *LHX2*) and **(C)** off-target gene markers (*PAX3*, *DLX4*, *EYA3*, *SOX3*) that are gradually increased or decreased during *in vitro* differentiation of hiPSCs to hOPCs.

In addition to embryonic HC markers, we also explored our differentiating cultures for the expression of genes characteristic of other epithelial cells in our culture differentiation system. We detected a progressive increase from day 0 to day 13 *in vitro* in the expression level of a subset of known inner ear cell supporting gene markers (i.e., *PROX1*, *EGFR*, *LGR5‥*; Figure 8A). There was also evidence that at least some neuronal differentiation was taking place under our culture conditions. This differentiation principally took place between day 6 and day 13 *in vitro* as revealed by the expression of a subset of genes (i.e., *NEUROD1*, *NEUROG1‥*) characteristic of neuronal cell types in the native developing inner ear (Figure 8B). Nevertheless, in addition to otic lineage cells, we also noticed an occurrence of some off-target cell gene markers belonging to other lineage (e.g., *PAX3*, *DLX4*) that promote anterior placode phenotype (Figure 8C). Other control non-otic genes were expressed at a lower level and their expression remained stable during the *in vitro* differentiation (i.e., *SOX3*, *DLX4* and *EYA4*), while the expression of mesoderm and endodermal lineage gene markers were undetectable in differentiated cell cultures at day 13. Taken together, and even though our differentiating protocol led to a high yield of cells displaying otic-like transcriptomic profiles, these data also indicate some kind of cell heterogeneity and/or phenotypic immaturity inside our cultures. Interestingly, as it can be found in the *in vivo* situation, several components of signaling pathways including WNT (e.g., *WNT5A*, *WNT7B*), SHH (e.g., *GLI3*, *HHIP*) and NOTCH (e.g., *JAG1*, *HES1*) were found to be upregulated during the time course of *vitro* guidance of hiPCs toward early otic/placodal cells as well as their subsequent specifications to hOSPCs populations (Supplementary Figures S2–S4). Other components of the TGF-β signaling pathway, such as Transforming Growth Factor Beta-Receptor (TGFβ-R) were also active during the *in vitro* guidance phase of hOSPCs enrichment from hiPSCs (Supplementary Figure S5). Altogether these results support a dual modulation of TGF-β/WNT pathways under exposition to FGF3/10 as an effective method for the derivation of cell populations that display rapid and transient expression of early otic/placodal markers followed by late hOSPCs markers.

## Discussion

Stem cell-based cell therapy raises many expectations for treating disorders caused by cell loss and are now possible through advances in relevant *in vitro* strategies for the production of cell progenitors (Grabel, 2012). Among numerous possible examples, attempts for the treatment of retinal degeneration (Lamba et al., 2009; Schwartz et al., 2012) or Parkinson’s disease (Grealish et al., 2014) with transplantation of stem cell byproducts have established proofs-of concept for the great interest of such approaches. Although several remaining challenges exist, replacement of lost sensory HCs has increasingly become a plausible strategy for the treatment of balance and hearing disorders (Martinez-Monedero and Edge, 2007; Géléoc and Holt, 2014). The inner ear is a promising system to develop and apply cell-based therapies, however, the availability of a renewable source of OSPCs is a major prerequisite. Whereas *in vitro* guidance protocols for the differentiation of HC-like cells from somatic tissue or PSCs are continuously evolving (Koehler et al., 2013, 2017; Ronaghi et al., 2014; Costa et al., 2015; McLean et al., 2017), the generation of human OSPCs remains a challenging endeavor. The lack of individual specific otic lineage markers represents another difficulty that could hamper the identification of OSPCs (Dincer et al., 2013; Ealy et al., 2016). In this study, our aim was to devise a new protocol allowing the differentiation of a high yield of human OSPC-like cells from hiPSCs by systematically screening factors and signals that can promote OSPC lineage path. We used qPCR and immunocytochemistry approaches to track the emergence of NNE, PPE, OP and OSPCs-like phenotypes by monitoring the expression of a panel of known lineage gene markers. In addition, RNA-Seq transcriptomic data was used to validate developmental path towards human OSPC and embryonic HC phenotypes. We found that during the initial step of otic induction from hiPSCs, inhibition of TGF-β and WNT pathways under continuous exposure to FGF3/10 contribute to a robust differentiation of hiPSCs into the NNE/PPE and OP marker-expressing cells. We noticed a significant upregulation of a panel of NNE/PPE and OP stage markers in cells treated with FGF3/10 along with TGF-β/WNT inhibitors (i.e., FSBD cultures). In addition, we found an enrichment of OP markers, i.e., about 55% of PAX2 immuno+ cells in FSBD treated-cultures at day 6. This % of PAX2 immuno+ cells generated in this short-term culture paradigm yielded a significant higher level of human otic induction under FSBD culture condition when compared to our previous otic differentiation protocol (Lahlou et al., 2018). Indeed, we previously reported that, using the same hiPSCs, treatment of cells with FGF3/10 only resulted in ~14% of PAX2 immuno+ cells at day 6 *in vitro*. Moreover, previous works from other groups have reported similar efficacies of differentiation. For example, and using otic induction from hESC monolayer cultures by applying differentiating conditions that included retinoic acid, it was previously reported the generation of about 14% of PAX2 immuno+ cells at day 18 post-differentiation (Ealy et al., 2016). These data suggest that our newly defined protocol surpasses previous protocols, including ours, by its capacity to differentiate higher yield of PAX2 immuno+ OP cells from hiPSCs. Even though data from other groups have contributed to highlight the importance of FGF pathway activation to induce otic progenitor cells *in vitro* (Chen et al., 2012; Ronaghi et al., 2014); our study shed new lights on the power of a concomitant inhibition of WNT and TGF-β signaling, along with FGF3/10 treatment, that potentiates otic/placodal differentiation.

Of interest, TGF-β/WNT inhibition in FGF-exposed cultures was sufficient to decrease the expression of anterior placode (i.e., *PAX6*) and to promote expression of posterior placode (i.e., *PAX2/8*, *OTX1*, *SOX9*) gene markers. This requirement for progressive decrease of anterior placode lineage and FGF activation for proper ability to expand posterior placode (i.e., PAX2 immuno+ cells) has been previously shown in the developing mice (Chen and Streit, 2013; Dincer et al., 2013). In addition to the substantial generation of otic/placodal cells with our induction strategy, this is the first report showing the expression of embryonic HC markers (POU4F3 and MYO7A) as early as day 6 *in vitro*. The qPCR and RNA-seq analyses demonstrated an increase in the expression of these embryonic HC markers during the second phase of the *in vitro* guidance procedure. In addition, our protocol led to the upregulation of Notch ligands such as *DLL1* and *JAG2*, both known as embryonic HC markers. Of interest, a previous *in vivo* work demonstrated that *DLL1* and *JAG2* act together in a cooperative manner to regulate embryonic HC development in mammalian inner ear (Lewis et al., 1998; Lanford et al., 1999; Zine et al., 2000, 2014; Kiernan et al., 2005a). Furthermore, gene expression analysis revealed that our differentiation protocol results in the production of a cell population with multiple phenotypes (i.e., NNE/PPE and OP) at day 6. However, it is not possible to ascertain whether the occurrence of such cell populations may reflect the *in vivo* situation. In this line, a recent study, using a single cell trajectory analysis revealed the generation of cells with mixed phenotypes in monolayer differentiated hESC cultures (Ealy et al., 2016). This may suggest that the use of a monolayer culture system combined with suppression of mesendoderm lineage under FGF exposition creates a permissive environment for the production of different otic/placodal cell populations. The early induction of a subset of embryonic HC markers at day 6 of hiPSC *in vitro* differentiation suggested that differentiated otic/placodal progenitors remained competent to respond to initial HC determination cues during the second phase of the differentiation.

The FGF pathway has been shown to be involved in the full program of the *in vivo* otic territory specification during inner ear embryogenesis (Pirvola et al., 2000; Zelarayan et al., 2007; Yang et al., 2013). In line with this, our results showed that FGF pathway activity contributes to otic/placodal induction process, but is not sufficient alone to induce efficient transition of hiPSCs towards a more advanced otic lineage. Our data revealed that activation of FGF signal transduction needs to be temporally accompanied with inhibition of WNT/TGF-β pathways, early during the differentiation process, for an optimal otic induction from hiPSCs. Our data suggest a functional interplay between FGF, WNT TGF-β signaling pathway modulations to initiate an efficient *in vitro* differentiation. To further explore the role of WNT, we reasoned that similar to the *in vivo* situation with the multiple functions of WNT pathway (Ohyama et al., 2006; Jayasena et al., 2008), its activation could promote initial HC lineage. To test this hypothesis, we challenged the generated FSBD early otic/placodal cells with WNT3A from day 6 to day 13. We found that many transcripts involved in the canonical WNT/β-catenin pathway were expressed at day 13 of differentiation such as, *WNT5A*, *WNT5B*, *WNT6* and *WNT7B* suggesting that these critical cell fate regulators are active at this stage of *in vitro* differentiation. Interestingly, these multiple WNT pathway components have previously shown to be expressed in the mouse early embryonic day E8–E11.5 otocyst (Atkinson et al., 2015; Geng et al., 2016). In addition, a previous work using microarray analysis reported an upregulation of WNT5A in dissected inner ear tissue from mouse developmental stages E9–E15 (Sajan et al., 2007). A similar finding on the effect of WNT (i.e., WNT3A) in inner ear embryonic patterning was described in the zebrafish (Forristall et al., 2014). There is also evidence that WNT signaling is required for the specification of OP size by restricting the otic lineage to a subset of PAX2+ otic/placodal cells “en route” to form the otocyst/vesicle sensory cell types (Freyer and Morrow, 2010). In line with these evidences, it is reasonable to hypothesize that PAX2+ cells induced at day 6 maintained otic identity and promoted the expression of embryonic HC markers, such as MYO7A, DLL1, JAG2 under WNT activation. Furthermore, the WNT pathway is known to regulate both proliferation and differentiation in a wide variety of biological systems (MacDonald et al., 2009; van Amerongen and Nusse, 2009). During inner ear development, WNT co-operates with other signaling pathways, particularly NOTCH and FGF to specify OP and its sensory derivatives (Munnamalai and Fekete, 2013). Our findings suggest that the main role of FGF activity and WNT/TGF-β inhibition, during inner ear induction, is to activate a transcriptional network, which in turn may be sufficient to implement embryonic HC program autonomously. In line with this hypothesis, a recent *in vitro* work with chick embryos (Anwar et al., 2017) suggested that downstream of FGF pathway a few transcription factors form a circuit of positive feedback loops that is sufficient to maintain otic progenitor identity and thus kept cells competent to respond to another signaling input.

In conclusion, our study demonstrates that WNT/TGF-β pathways inhibition under continuous FGF activation is a novel and rapid guidance strategy for efficient induction of otic progenitors from hiPSCs and establish the conditions for their subsequent differentiation to OSPCs/embryonic HCs. Beside the generation of enriched human OSPCs in these conditions, the method reveals a level of heterogeneity that may be useful in identifying important additional modifications in *in vitro* conditions to selectively stabilize the developmental otic sensory trajectory lineage. Advances in knowledge of stages and time course of OSPC differentiation offers an opportunity to study fundamental questions of human inner ear development, in addition to the use of their derivatives for testing drugs and cell therapy for deafness and balance disorders caused by HC loss.

## Author Contributions

HL, AL-J and AF performed the experiments. HL, EN, SA and AZ analyzed the data. AZ wrote the main manuscript text. All authors reviewed the manuscript.

## Conflict of Interest Statement

The authors declare that the research was conducted in the absence of any commercial or financial relationships that could be construed as a potential conflict of interest.

## Footnotes

^1^https://www.qiagenbioinformatics.com/products/ingenuity-pathway-analysis

## Supplementary Material

FIGURE S1 Generation of otic/placodal progenitor cells at day 6 of differentiation. Early differentiating SOX2 and PAX2 immuno-positive cells from hiPSCs in FGF3 + FGF10 **(A)** and FSBD **(B)** cultures. In FSBD-treated cultures, we observed a large population of SOX2 (shown in green) and PAX2 (shown in white) immuno-positive when compared to FGF3/10-treated cultures. Coexpression of otic markers PAX2 and SOX2 was observed in a subset of differentiated cells in FGF3/10 and FSBD treated cultures. Cell nuclei were counterstained with Hoechst (blue). Scale bars = 20 μm.

FIGURE S2 Functional network analysis related WNT gene markers upregulated during the time course of *in vitro* otic induction. **(A)** The most significant IPA network assembled around WNT in day 13 cultures showing the network of WNT and their close interactions with SOX9 and Hedgehog genes. Genes upregulated included in the day 13 signature are in pink and those downregulated in the signature are in green. Continuous lines between nodes indicate direct molecular interactions between connected transcripts and dotted lines indicate indirect functional interactions between transcripts. **(B)** The figure shows read numbers of four selected WNT gene (*WNT5A*, *WNT5B*, *WNT6* and *WNT7B*) ligands that are progressively enriched in day 13 differentiated cultures.

FIGURE S3 Functional network analysis related to day 6 and day 13 up-regulated genes. **(A)** Characterization of Sonic Hedgehog signaling pathway during *in vitro* differentiation of hiPSCs to hOPCs. Genes shown in pink are up-regulated in day 6 and day 13 and in green are downregulated genes. **(B)** Figure shows read numbers of Sonic Hedgehog pathway related genes (*HHIP*, *GLI3*, *SMO*, *DYRK1*) that are gradually increased during *in vitro* differentiation of hiPSCs.

FIGURE S4 Significant network and genes assembled around NOTCH pathway. **(A)** Gene interaction networks were constructed using the IPA software. Nodes shaded in pink represent genes that are upregulated in day 6 and day 13, and green nodes are genes that are downregulated in day 6 and day 13 cultures. These networks assembled by up- and down-regulated genes include genes linked to Notch signaling pathway. The intensity of the node color indicates the degree of gene up-regulation or downregulation. Edges (lines) and nodes are annotated with labels that illustrate the nature of the relationship between genes and their functions. A solid line represents a direct interaction and a dotted line an indirect interaction. **(B)** Figure shows read numbers of Notch pathway genes (*NOTCH3*, *JAG1*, *HES1*, *HES5*, *HEY1*, *HEY2*) that are gradually increased or decreased during the time course of *in vitro* differentiation.

FIGURE S5 Ingenuity pathway analysis showing WNT and TGF-β pathway components up-regulated in day 13 signature. The color intensity indicates their degree of upregulation. Downregulated genes are shown in green and upregulated genes are shown in pink. Uncolored genes were identified as not differentially expressed in our analysis. The deregulated genes were imported into IPA and each gene identifier was overlaid onto a global molecular network developed from information contained in the Ingenuity Pathways Knowledge Base. IPA, Ingenuity pathway analysis software (http://www.ingenuity.com).

TABLE S1 List of gene-specific primers used for RT-qPCR for gene expression.

TABLE S2 List of primary and secondary antibodies used for immunohistochemistry.
