# Supplementary figures and images for: Enriched Differentiation of Human Otic Sensory Progenitor Cells Derived From Induced Pluripotent Stem Cells
Source: Front Mol Neurosci. 2018 Dec 20;11:452. doi: 10.3389/fnmol.2018.00452 (PMC6306956; doi:10.3389/fnmol.2018.00452)

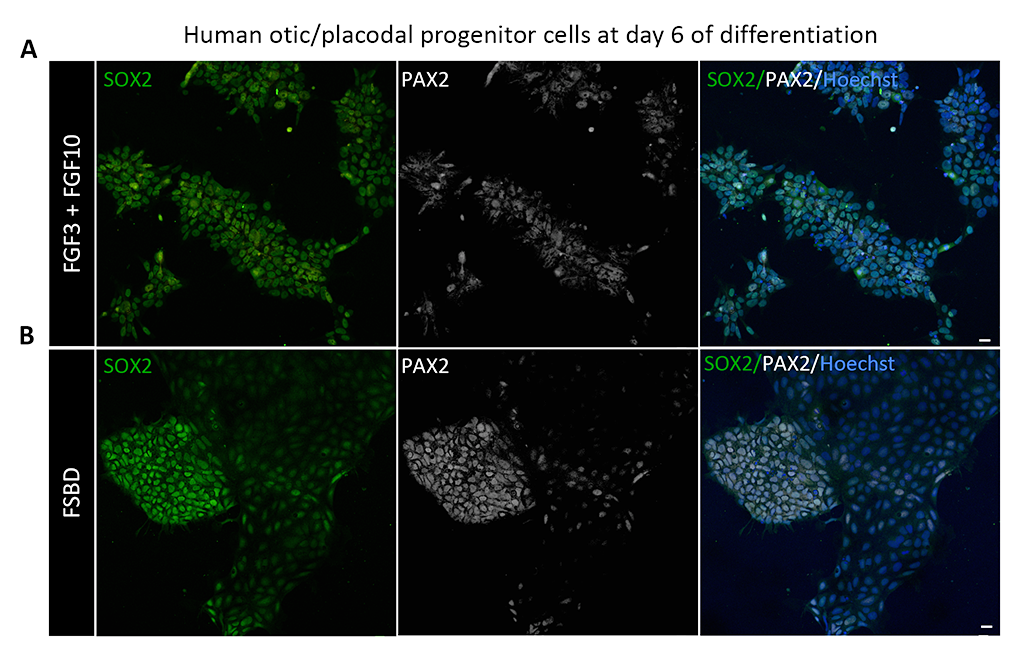

Supplement: FIGURE S1 — Generation of otic/placodal progenitor cells at day 6 of differentiation. Early differentiating SOX2 and PAX2 immuno-positive cells from hiPSCs in FGF3 + FGF10 (A) and FSBD (B) cultures. In FSBD-treated cultures, we observed a large population of SOX2 (shown in green) and PAX2 (shown in white) immuno-positive when compared to FGF3/10-treated cultures. Coexpression of otic markers PAX2 and SOX2 was observed in a subset of differentiated cells in FGF3/10 and FSBD treated cultures. Cell nuclei were counterstained with Hoechst (blue). Scale bars = 20 μm. [file Image_1.TIF]

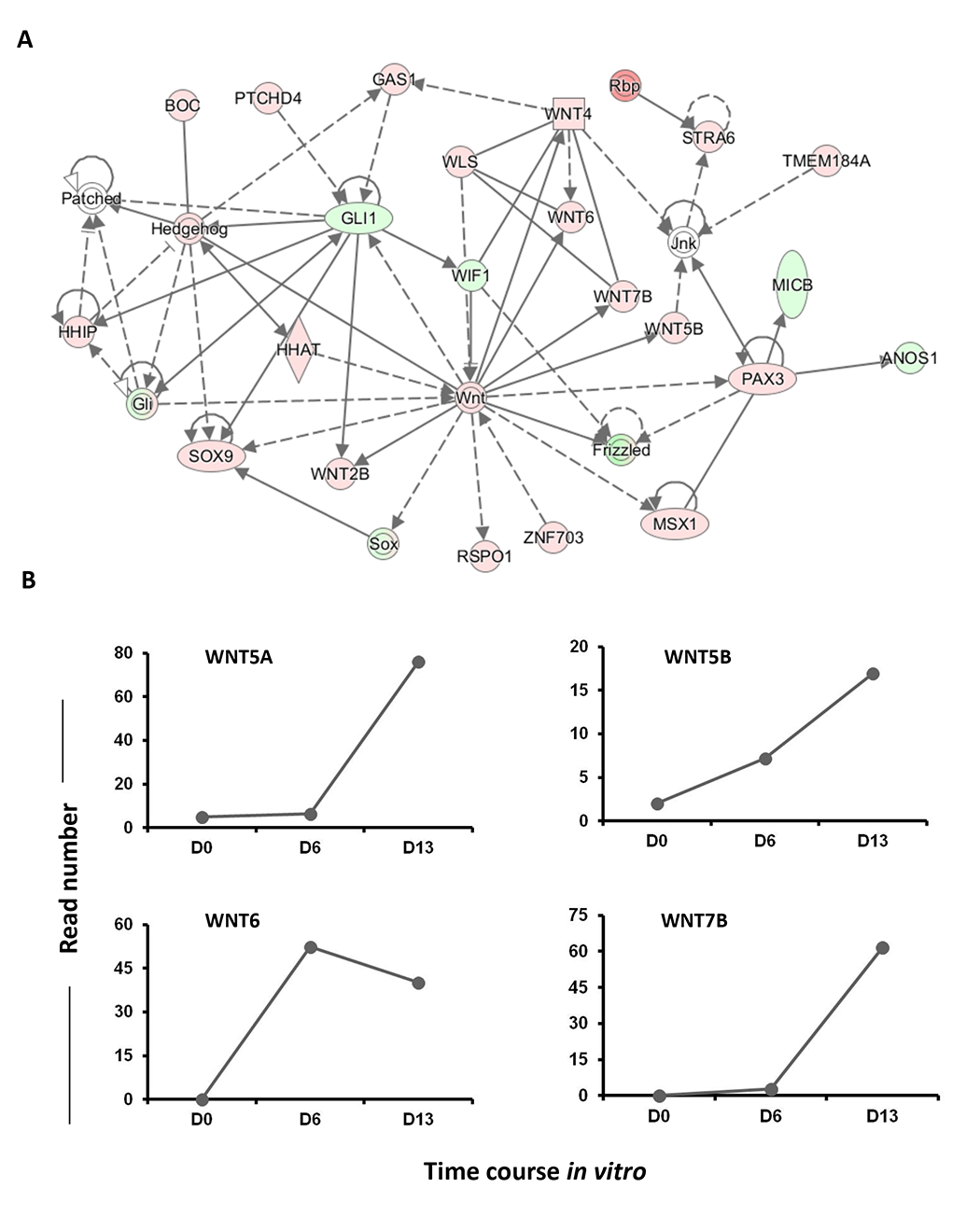

Supplement: FIGURE S2 — Functional network analysis related WNT gene markers upregulated during the time course of in vitro otic induction. (A) The most significant IPA network assembled around WNT in day 13 cultures showing the network of WNT and their close interactions with SOX9 and Hedgehog genes. Genes upregulated included in the day 13 signature are in pink and those downregulated in the signature are in green. Continuous lines between nodes indicate direct molecular interactions between connected transcripts and dotted lines indicate indirect functional interactions between transcripts. (B) The figure shows read numbers of four selected WNT gene (WNT5A, WNT5B, WNT6 and WNT7B) ligands that are progressively enriched in day 13 differentiated cultures. [file Image_2.TIF]

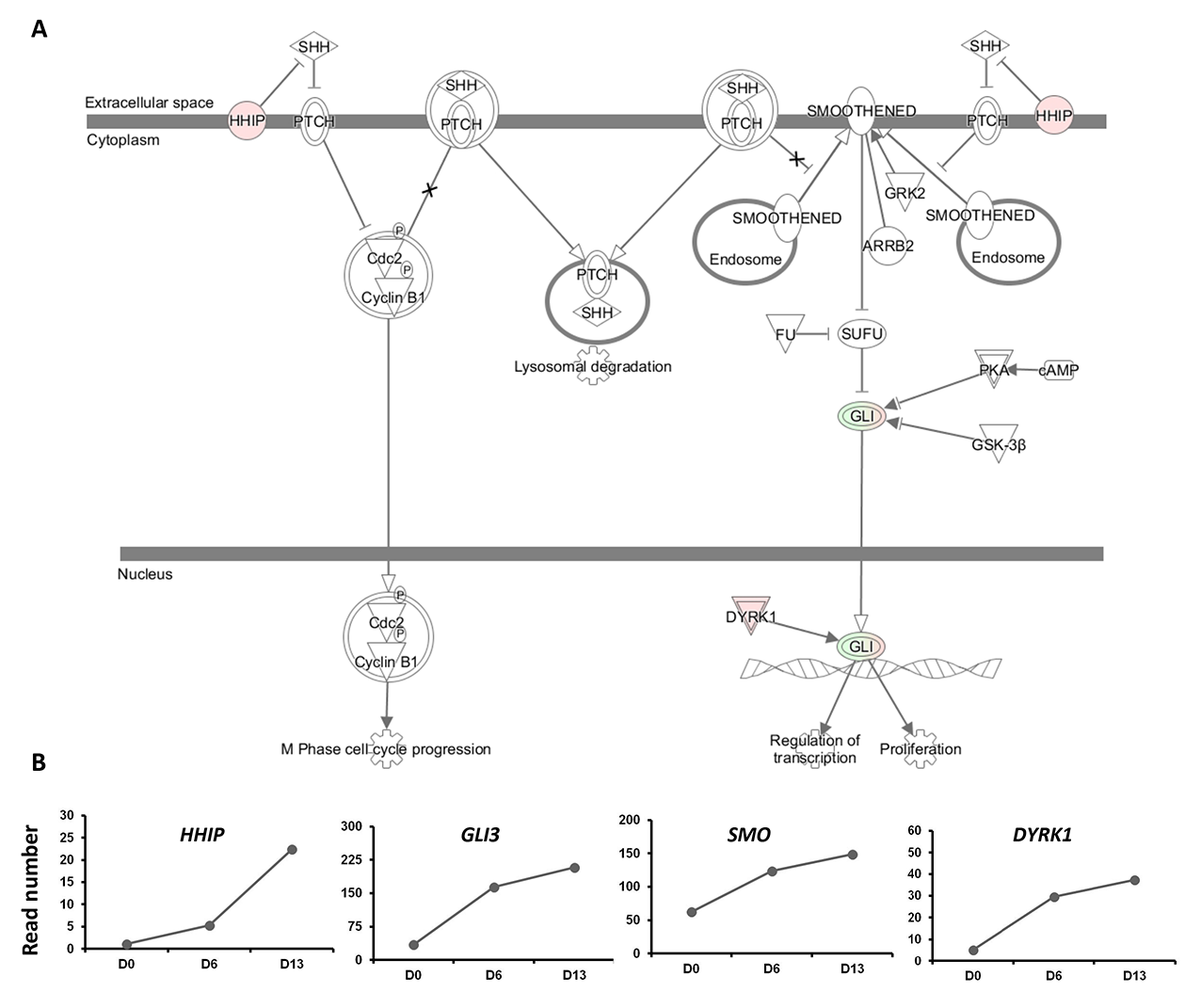

Supplement: FIGURE S3 — Functional network analysis related to day 6 and day 13 up-regulated genes. (A) Characterization of Sonic Hedgehog signaling pathway during in vitro differentiation of hiPSCs to hOPCs. Genes shown in pink are up-regulated in day 6 and day 13 and in green are downregulated genes. (B) Figure shows read numbers of Sonic Hedgehog pathway related genes (HHIP, GLI3, SMO, DYRK1) that are gradually increased during in vitro differentiation of hiPSCs. [file Image_3.TIF]

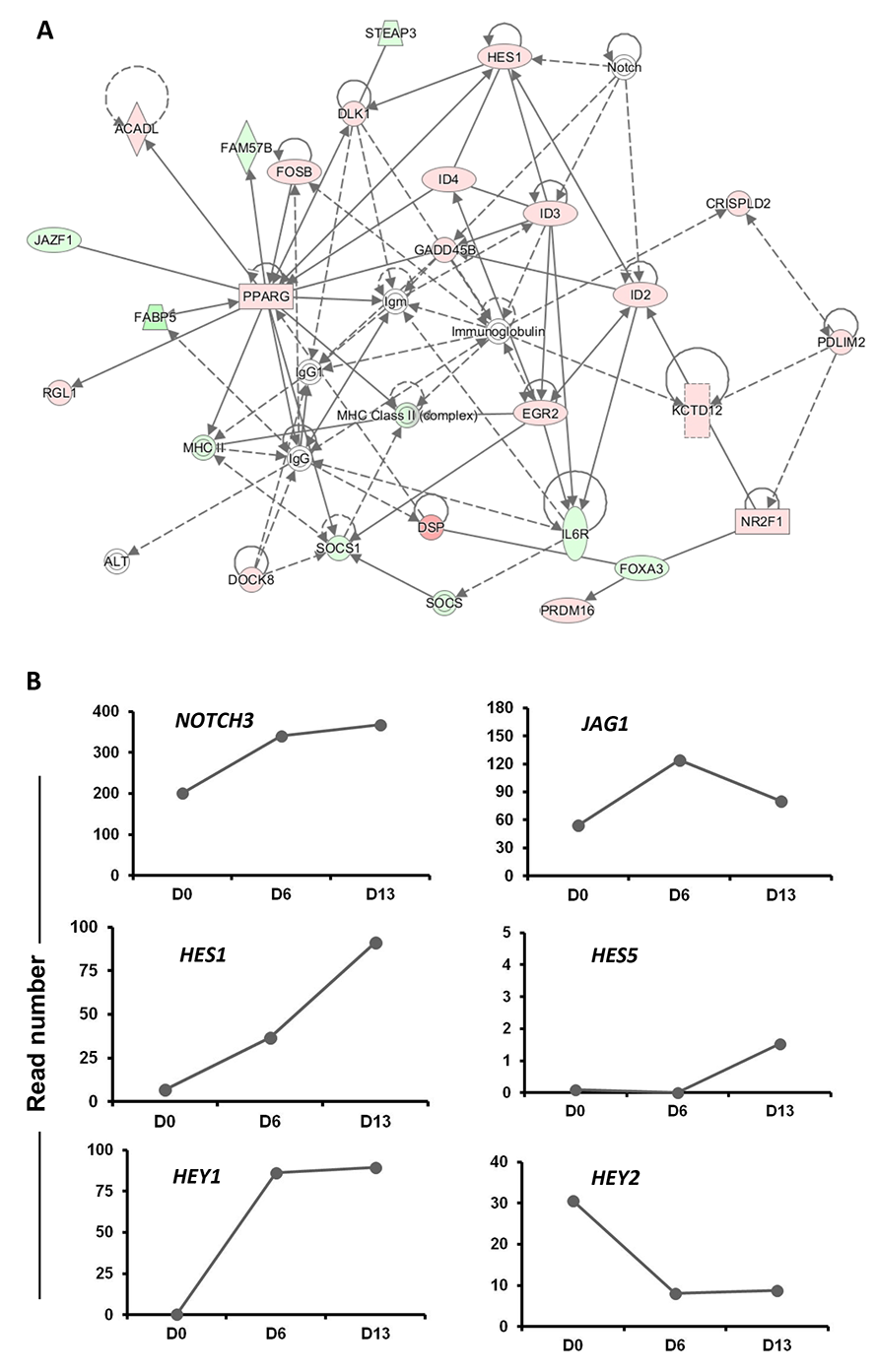

Supplement: FIGURE S4 — Significant network and genes assembled around NOTCH pathway. (A) Gene interaction networks were constructed using the IPA software. Nodes shaded in pink represent genes that are upregulated in day 6 and day 13, and green nodes are genes that are downregulated in day 6 and day 13 cultures. These networks assembled by up- and down-regulated genes include genes linked to Notch signaling pathway. The intensity of the node color indicates the degree of gene up-regulation or downregulation. Edges (lines) and nodes are annotated with labels that illustrate the nature of the relationship between genes and their functions. A solid line represents a direct interaction and a dotted line an indirect interaction. (B) Figure shows read numbers of Notch pathway genes (NOTCH3, JAG1, HES1, HES5, HEY1, HEY2) that are gradually increased or decreased during the time course of in vitro differentiation. [file Image_4.TIF]

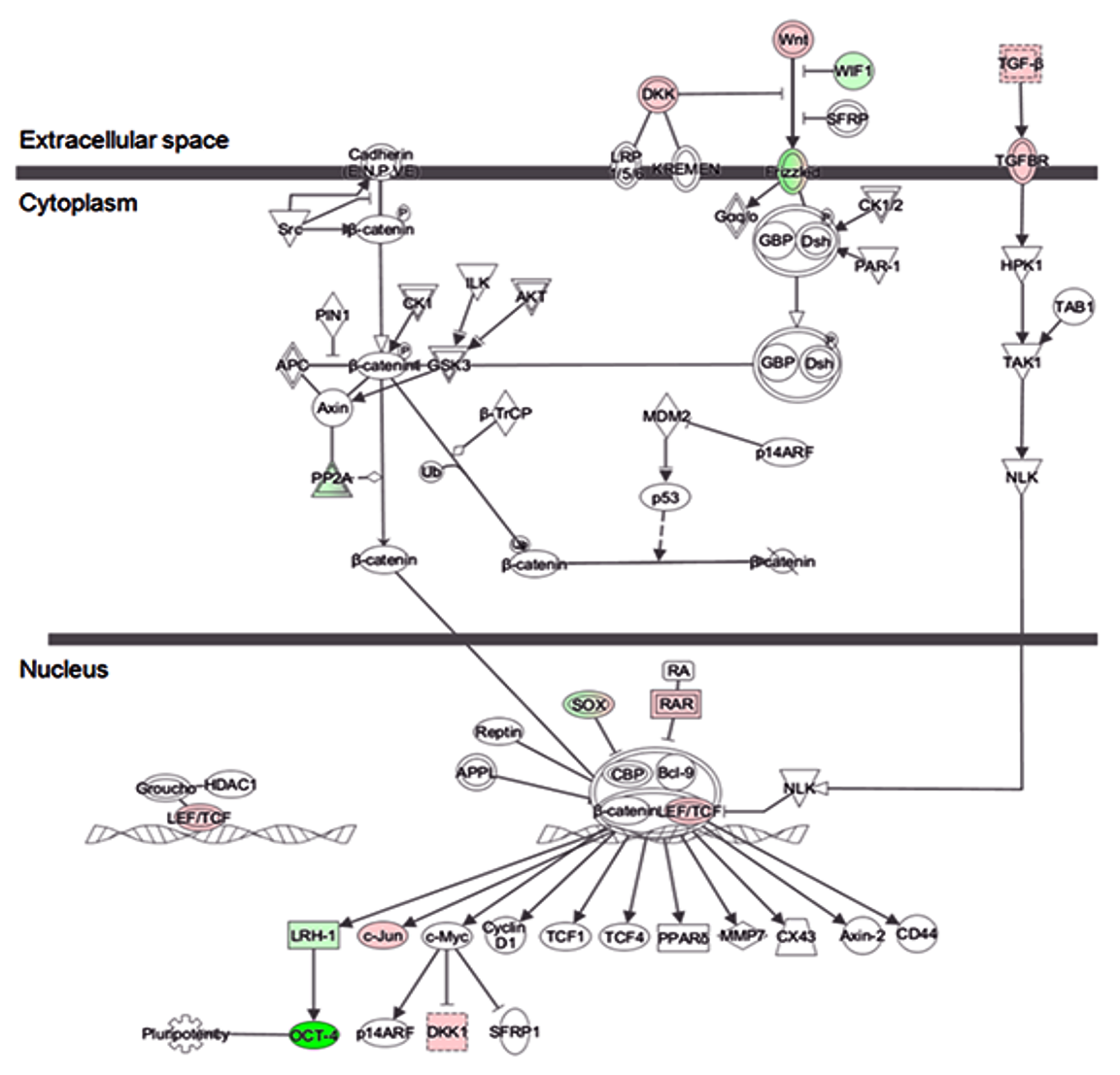

Supplement: FIGURE S5 — Ingenuity pathway analysis showing WNT and TGF-β pathway components up-regulated in day 13 signature. The color intensity indicates their degree of upregulation. Downregulated genes are shown in green and upregulated genes are shown in pink. Uncolored genes were identified as not differentially expressed in our analysis. The deregulated genes were imported into IPA and each gene identifier was overlaid onto a global molecular network developed from information contained in the Ingenuity Pathways Knowledge Base. IPA, Ingenuity pathway analysis software (http://www.ingenuity.com). [file Image_5.TIF]
