# Supplementary material for: Enriched Differentiation of Human Otic Sensory Progenitor Cells Derived From Induced Pluripotent Stem Cells
Source: Front Mol Neurosci. 2018 Dec 20;11:452. doi: 10.3389/fnmol.2018.00452 (PMC6306956; doi:10.3389/fnmol.2018.00452)
Supplement: TABLE S1 — List of gene-specific primers used for RT-qPCR for gene expression. [file Data_Sheet_1.docx]

**Supporting Table S1** List of gene-specific primers used for RT-qPCR for gene expression

| **Gene** | **FP** | **RP** |
| --- | --- | --- |
| *GAPDH* | ACACCATGGGGAAGGTGAAG | GTGACCAGGCGCCCAATA |
| *DACH1* | GAGAAGCAGTTGGCTATGGAAC | ACGCCGTTTCGTCTCAAAC |
| *DLX3* | AGCCTCCTACCGGCAATAC | TTCCGGCTCCTCCTTCAC |
| *DLX5* | GCTAGCTCCTACCACCAGTAC | GGTTTGCCATTCACCATTCTCA |
| *DLX6* | CAGCTTCCTTAGGACTGACACA | GAGGATTACTGCCCTGCTTCA |
| *EMX2* | GCCCCATAAATCCGTTCCTCA | CAAGTCCGGGTTGGAGTAGAC |
| *EYA1* | CCGCTACAGACGGGTAAAAG | TTCCCTCTTAGCTGGACCAA |
| *EYA2* | ACCAGATCCACGTTGATGAC | CGTCAGCGGAGAAGTTGTA |
| *FOXI1* | GACAAGCGCCTCACTCTCA | CCGGCCTTGCTCTTGTTGTA |
| *FOXI3* | TCGCTCAACGACTGCTTCAA | GCAGTTCGGATCAAGAGTCCAA |
| *GATA2* | CAGAACCGACCACTCATCAA | CACAGGCGTTGCAGACAG |
| *GATA3* | CACGGTGCAGAGGTACCC | AGGGTAGGGATCCATGAAGCA |
| *MSX1* | CGCAGGTGAAGATATGGTTCC | CTCCAGCTCTGCCTCTTGTA |
| *OTX1* | GACCTCCTGCACCCATCC | CAGCTGTGAACGCGTGAA |
| *PAX2* | CGGCTGTGTCAGCAAAATCC | GCTTGGAGCCACCGATCA |
| *PAX6* | TTGCCCGAGAAAGACTAGCA | TCTCCATTTGGCCCTTCGATTA |
| *PAX8* | GCCCAGTGTCAGCTCCATTA | GCTGTCCATAGGGAGGTTGAA |
| *SIX1* | GGTTTAAGAACCGGAGGCAAA | TGCTTGTTGGAGGAGGAGTTA |
| *SOX9* | AACGCCGAGCTCAGCAA | CGCTTCTCGCTCTCGTTCA |
| *TBX2* | CCAACAACATCTCTGACAAGCA | TTGGCTCGCACTATGTGGAA |
| *TFAP2A* | TAAAGCTGCCAACGTTACCC | GCACACGTACCCAAAGTCC |
| *ATOH1* | GCAATGTTATCCCGTCGTTCAA | TCGGACAAGGCGTTGATGTA |
| *AQP1* | GCCATCGGCCTCTCTGTA | AAAGGACCGAGCAGGGTTAA |
| *CHRNA10* | GCGCTCACCGTCTTCC | ACCACAGTAATGCAGGTTCA |
| *JAG2* | CTGGGTGGAAGACTGCAACA | AGCAGACAAGGCTTCCATCC |
| *LBH* | ATGGAGGAGATCGGCCTCA | AAAATCTGACGGGTCTGGGAA |
| *MYO15A* | GAAACCACTGTGCTGTCCAA | ACCAGGATGCTCCCAATGTA |
| *MYO3A* | GGCAACTGAACACCAGATTGAC | CTGCCCGAATGCAAAGCAAA |
| *MYO6* | GTGCTGGTGCTTCTGAAGATA | CTAGTGCAGCCTCGGTTTAA |
| *MYO7A* | TGAGACCCAGTTTGGCATCA | GGTGTCTCGGTTCTTCTCCA |
| *OTOF* | CCTGCAGATCTGGGATGC | ATCTCCATGGTGCACTGCTT |
| *POU3F4* | CACCACTCACCGCACACTA | TTGGCCGCTTGACGTGATA |
| *POU4F3* | CTGCAAGAACCCAAATTCTCCA | GGCTCTCATCAAAGCTTCCAAA |
| *TMC4* | CTTTACTGCCCCTGCTTAACAC | AGCAGGTGGAGAAGAGGGTA |

**Supporting Table S2** List of primary and secondary antibodies used for immunohistochemistry

|  | **Host** | **Dilution** | **Provider** |
| --- | --- | --- | --- |
| **DLX5** | goat | 1 : 50 | Santa-Cruz |
| **GATA3** | mouse | 1 : 50 | Santa-Cruz |
| **MYO7A** | rabbit | 1 : 200 | Proteus (Coger) |
| **POU4F3** | mouse | 1 : 100 | Abnova |
| **PAX2** | rabbit | 1 : 100 | Covance |
| **SOX2** | goat | 1 :150 | Santa Cruz |
| **PAX8** | goat | 1 : 100 | Covance |
| **Alexa fluor 594** | donkey anti-mouse | 1 : 500 | Molecular Probes |
| **Alexa fluor 594** | donkey anti-goat | 1 : 500 | Molecular Probes |
| **Alexa fluor 488** | donkey anti-mouse | 1 : 500 | Molecular Probes |
| **Alexa fluor 488** | donkey anti-goat | 1 : 500 | Molecular Probes |
| **Alexa fluor 488** | donkey anti-rabbit | 1 : 500 | Molecular Probes |
